# Reconfigurable Transmission Design for PASS-MIMO via Waveguide Indexing

**DOI:** 10.3390/s26144407

**Published:** 2026-07-11

**Authors:** Yaxian Wang, Songjie Yang, Juhong Peng

**Affiliations:** 1 Manchester Metropolitan Joint Institute, Hubei University, Wuhan 430062, China; 21906317@stu.mmu.ac.uk; 2 National Key Laboratory of Wireless Communications, University of Electronic Science and Technology of China, Chengdu 611731, China; yangsongjie@std.uestc.edu.cn; 3 School of Artificial Intelligence, Hubei University, Wuhan 430062, China

**Keywords:** pinching-antenna system, multiple-input multiple-output, waveguide, index modulation, generalized spatial modulation, beamforming

## Abstract

To address the stringent requirements of 6G industrial Internet of Things (IoT) and ultra-dense networks on spectral efficiency, hardware cost, and transmission reliability, this paper investigates waveguide index modulation based on the pinching-antenna system (PASS), a promising flexible multiple-input multiple-output (MIMO) architecture featuring large-scale reconfigurability and robust line-of-sight (LoS) link establishment. A two-stage sparse transmission framework is proposed, where a Simulated Annealing-based Constrained Discrete Optimization (SA-CDO) algorithm is first employed to optimize pinching-antenna (PA) positions and construct a near-orthogonal equivalent channel dictionary for inter-waveguide interference suppression. Subsequently, an Orthogonal Least Squares-based Constellation-Constrained (OLS-CC) detector is developed to jointly recover active waveguide indices and modulation symbols with low computational complexity. Monte Carlo simulations demonstrate that the proposed scheme consistently outperforms conventional antenna index modulation under both LoS and Rician fading channels across the entire SNR range. The SA-CDO optimization significantly reduces the bit error rate (BER), while the OLS-CC detector further improves sparse recovery accuracy and reduces the detection complexity from exponential to polynomial order. These results provide valuable insights for the design of highly reliable 6G IoT communication systems.

## 1. Introduction

With the rapid evolution of sixth-generation (6G) wireless communications, emerging applications such as the industrial Internet of Things (IIoT), integrated sensing and communication (ISAC), and ultra-dense wireless networks are imposing increasingly stringent requirements on spectral efficiency, hardware efficiency, transmission reliability, and intelligent resource management [1,2]. These emerging application scenarios require communication architectures that can simultaneously provide high flexibility, low hardware cost, and robust transmission performance, thereby motivating the development of reconfigurable multiple-input multiple-output (MIMO) technologies. Traditional MIMO systems exploit spatial multiplexing and diversity gains through fixed-antenna arrays and have become a fundamental enabler of high-data-rate wireless communications. However, conventional fixed-antenna MIMO architectures suffer from several inherent drawbacks. Since each antenna element requires a dedicated radio-frequency (RF) chain, hardware cost and power consumption increase significantly with system scale [3]. Moreover, the fixed-antenna configuration cannot adapt to dynamic propagation environments, limiting achievable beamforming gains and transmission reliability, which has motivated the development of flexible MIMO architectures [4]. By dynamically reconfiguring antenna positions, radiation patterns, or propagation environments, these architectures can adapt transmission strategies to channel variations. Representative paradigms include fluid antenna systems (FAS) [5], movable antenna systems, and reconfigurable intelligent surface (RIS)-assisted MIMO [6]. FAS enables channel reshaping through continuous antenna position adjustment, while movable antenna systems exploit spatial degrees of freedom to enhance signal quality and suppress interference. Nevertheless, practical deployment challenges remain. FAS requires additional position-control or switching mechanisms, movable antenna systems rely on mechanical displacement with limited mobility and reliability, and RIS-assisted systems provide only passive beamforming gains without active signal generation or amplification.

Against this background, the pinching-antenna system (PASS) has recently gained considerable attention from academia and industry as a novel and cost-effective flexible MIMO paradigm due to its distinctive advantages of large-scale reconfigurability, robust Line-of-Sight (LoS) link construction capability, excellent scalability, and reliable near-field communication support [7,8,9]. Unlike traditional fixed-antenna arrays that suffer from rigid configuration and limited adaptability, PASS deploys pinching antennas (PAs) along dielectric waveguides, enabling flexible radiation pattern adjustment through dynamic position reconfiguration of the PAs. A unique fundamental feature of PASS is that multiple PAs on the same waveguide can be activated simultaneously without additional radio-frequency chains [10]. By dynamically optimizing PA locations for beamforming design, PASS effectively reduces propagation path loss and mitigates the adverse effects of both large-scale and small-scale fading, thereby offering a promising new approach to overcoming the inherent limitations of conventional multi-antenna systems [11,12]. Ref. [13] further quantified this performance advantage by deriving a closed-form upper bound on the array gain of PASS and proved that optimizing the number of PAs and their inter-spacing can achieve a significantly larger array gain than conventional fixed-antenna arrays. Furthermore, recent research works have further validated the superior performance of PASS. Ref. [14] achieved precise and flexible radiation control by adjusting the coupling spacing between PAs and optimizing beamforming strategies. Ref. [15] conducted an in-depth analysis on the multi-user capacity of PASS under discrete antenna activation and continuous PA sliding configurations. Ref. [16] proposed a hybrid PASS architecture that integrates mechanically reconfigurable PAs with electronically reconfigurable leaky-wave antennas. It combines the advantages of both technologies to mitigate the limitations of pure mechanical PASS systems. All these works further confirm PASS’s remarkable advantages over traditional multi-antenna architectures in supporting next-generation wireless communications. The comprehensive survey in [17] further consolidates these findings and identifies efficient index modulation integration and low-complexity transceiver design as two critical open research challenges for practical PASS deployment.

Index modulation (IM) has emerged as a promising technique to enhance spectral efficiency in wireless communications by uniquely mapping information bits onto discrete resource indices, thereby enabling additional information transmission without increasing bandwidth or transmit power [18]. Among the existing IM schemes, spatial modulation (SM) and its extended form, generalized spatial modulation (GSM), have attracted significant interest due to their ability to offer an attractive tradeoff between spectral efficiency and implementation complexity—balancing high data rate transmission with manageable hardware and computational overhead [19]. For multi-user SM systems, Ref. [20] proposed a polarization-aided detection and decoding scheme, which exploits the nested structure of polar codes to decode all user messages via a single polar decoder, and further designed an iterative polarization-aided scheme to eliminate inter-user interference, achieving superior error performance over traditional polar-coded SM systems. To adapt IM to the specific requirements of IoT communications, researchers have integrated IM with continuous phase modulation and spread-spectrum techniques, which effectively enhance distortion robustness and facilitate efficient multiuser access in resource-constrained IoT scenarios [21]. More importantly, recent work in [22] integrated IM with PASS, clearly demonstrating their complementary advantages: PASS provides flexible reconfigurability and beamforming capability, while IM contributes to improved spectral efficiency, laying a foundation for high-performance 6G IoT communications. However, the practical implementation of IM-based PASS systems still faces several critical challenges. First, the activation of index resources and the layout of PAs are tightly coupled, resulting in a high-dimensional nonconvex design problem that is computationally challenging to solve optimally [22,23]. Second, the detection complexity of IM schemes increases rapidly with the number of waveguides and PAs deployed, which severely limits their applicability in real-time communication scenarios that require low latency [7]. Meanwhile, the evolution of IM technology has progressed from traditional spatial modulation in fixed-antenna arrays to advanced reconfigurable architectures. For instance, position index modulation (PIM) in FAS jointly exploits the index domain and spatial domain by leveraging the dynamic position adjustment of fluid antennas, further improving transmission performance [24]. Ref. [25] extended this paradigm to MIMO scenarios, proposing a fluid antenna-assisted index modulation (FA-IM) scheme that maps information bits to both modulation symbols and fluid antenna position pattern indices and designing an optimized position codebook to maximize effective channel gains. Similarly, movable antenna-based IM schemes utilize the position degrees of freedom of antennas to optimize channel conditions, thereby enhancing the signal-to-noise ratio (SNR) and mitigating inter-user interference. Nevertheless, existing pinching-antenna index modulation (PA-IM) schemes are still constrained by their discrete antenna layouts and restricted mobility, which typically only allow small-scale position adjustments. This limitation hinders the global reconstruction of channel characteristics and the large-scale reshaping of radiation beams, ultimately restricting the performance improvement of IM in next-generation wireless communications.

In contrast, PASS enables continuous and flexible repositioning of PAs and large-scale deployment along dielectric waveguides. It effectively overcomes the inherent limitations of conventional discrete antenna arrays, including restricted mobility and rigid configurations, and creates a novel design space for IM that is specifically tailored to meet the diverse requirements of next-generation Internet of Things communications. Leveraging this unique capability, this paper proposes a two-stage waveguide-based index modulation transmission framework for PASS. In the first stage, a Simulated Annealing-based Constrained Discrete Optimization (SA-CDO) algorithm is developed to optimize PA deployment positions under practical physical constraints. This algorithm constructs a near-orthogonal equivalent channel dictionary and fundamentally suppresses inter-waveguide interference. In the second stage, an Orthogonal Least Squares-based Constellation-Constrained (OLS-CC) detector is designed at the receiver. It achieves low-complexity joint recovery of active waveguide indices and modulation symbols by decoupling sparse support recovery and constellation projection. The proposed framework exhibits strong robustness under both pure LoS propagation and practical Rician fading environments. Extensive Monte Carlo simulations demonstrate that the proposed scheme consistently outperforms conventional antenna index modulation schemes across all signal-to-noise ratio regimes. Specifically, SA-CDO-based PA position optimization reduces the bit error rate (BER) by more than an order of magnitude. Meanwhile, the OLS-CC detector further improves sparse recovery accuracy while maintaining polynomial-time complexity, which significantly enhances the overall system reliability.

The main contributions of this paper are summarized as follows.

1. A two-stage waveguide index modulation framework for PASS-MIMO is proposed. By treating dielectric waveguides as independent index units, the proposed framework decouples transmitter-side PA position optimization from receiver-side sparse signal detection, thereby effectively addressing the high-dimensional, non-convex optimization problem arising from the coupling between PA deployment and index activation.
2. A SA-CDO algorithm is developed for PA position optimization. Subject to practical constraints on waveguide boundaries and minimum PA spacing, the proposed algorithm sequentially optimizes the PA locations on each waveguide to construct a near-orthogonal equivalent channel dictionary, thereby substantially reducing inter-waveguide interference. Furthermore, the robustness of the proposed optimization strategy under Rician fading channels is theoretically analyzed and validated.
3. A low-complexity OLS-CC detector is proposed. By decoupling sparse support recovery from constellation projection, the detector reduces the computational complexity of joint detection from exponential to polynomial order while further improving the accuracy of joint index and symbol recovery by exploiting the near-orthogonal channel structure.

Extensive Monte Carlo simulations under both pure LoS and Rician fading channels demonstrate that the proposed scheme consistently outperforms the conventional fixed-PA deployment strategy across the entire SNR range. In particular, it achieves significant BER improvements while maintaining low computational complexity. The remainder of this paper is organized as follows. Section 2 introduces the system model of the proposed PASS-based waveguide index modulation framework. Section 3 presents the proposed SA-CDO optimization algorithm and the OLS-CC detector, together with their complexity and performance analyses. Simulation results are provided in Section 4, and conclusions are drawn in Section 5.

*Notations:* Bold lowercase and uppercase letters denote vectors and matrices, respectively. ${\left(\cdot \right)}^{T}$ and ${\left(\cdot \right)}^{H}$ represent the transpose and conjugate transpose. $I_{N}$ is the N×N identity matrix. ${∥\cdot ∥}_{F}$, ${∥\cdot ∥}_{2}$, and ${∥\cdot ∥}_{0}$ denote the Frobenius norm, $\ell_{2}$-norm, and $\ell_{0}$-pseudo-norm, respectively. tr(·) is the matrix trace. R and C represent real and complex number sets. $\mathrm{CN}(\mu ,\sigma^{2})$ denotes the circularly symmetric complex Gaussian distribution. ℜ(·) denotes the real part of a complex number. ⊙ denotes the Hadamard product. Blkdiag(·) denotes the block-diagonal matrix operator. *N*, *M*, $N_{r}$, and *K* denote the number of waveguides, pinching antennas per waveguide, receive antennas, and activated waveguides, respectively. $L_{\mathrm{wg}}$, λ, $n_{\mathrm{eff}}$, $d_{\mathrm{wg}}$, and $x_{R}$ denote the waveguide length, carrier wavelength, effective refractive index, inter-waveguide spacing, and receiver *x*-coordinate, respectively. H, G, and A denote the overall channel matrix, waveguide radiation matrix, and equivalent channel matrix, respectively. s, ξ, y, and n denote the sparse index-symbol vector, modulation symbol vector, received signal vector, and noise vector, respectively. X, M, and $C^{*}$ denote the PA position feasible set, modulation constellation set, and active waveguide index set, respectively.

## 2. System Model

**Figure 1:**
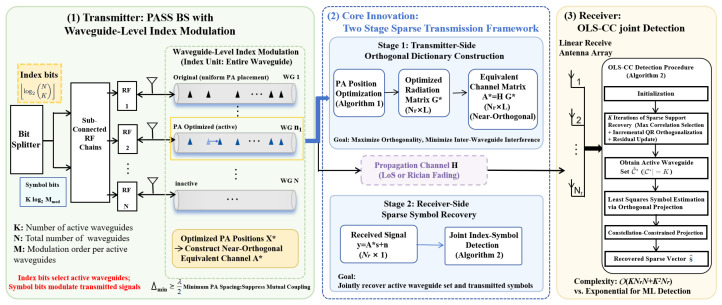
System architecture of the proposed two-stage waveguide-based index modulation scheme for PASS.

We consider a reconfigurable waveguide-based PASS architecture for index modulation, as illustrated in Figure 1. The system consists of a transmitter, a multi-waveguide PA array, and a one-dimensional linear receiver array. The transmitter adopts a sub-connected architecture, which is equipped with *N* RF chains and *N* parallel dielectric waveguides arranged along the *x*-axis. To ensure independent control of each waveguide, each RF chain is exclusively assigned to feed one waveguide, where the index of the waveguide is denoted as n∈{1,2,…,N}. Each dielectric waveguide has a fixed length of $L_{\mathrm{wg}}$ and is pre-equipped with *M* PAs that are uniformly distributed along the longitudinal direction of the waveguide in the initial configuration. The set of PA positions on the *n*-th waveguide is mathematically defined as

(1) $$X_{n}=\left\{x_{n1},x_{n2},\ldots ,x_{nM}\right\},$$

where each PA position $x_{nm}$ (corresponding to the *m*-th PA on the *n*-th waveguide) satisfies the boundary constraint

(2) $$x_{nm}\in \left[0,L_{\mathrm{wg}}\right],\;m\in \left\{1,2,\ldots ,M\right\}.$$

The feeding point of each waveguide is located at the origin of the *x*-axis, i.e., x=0. To suppress mutual coupling between adjacent PAs, which can degrade the radiation performance and channel quality, the spacing between two consecutive PAs on the same waveguide is constrained to be no less than the minimum spacing $\Delta_{\min}$, which is given by $\Delta_{\min}=\frac{\lambda }{2},$ where λ denotes the carrier wavelength. Thus, the PA spacing constraint can be explicitly expressed as

(3) $$x_{n(m+1)}-x_{nm}\geq \Delta_{\min}=\frac{\lambda }{2}.$$

In the initial configuration, the PAs are uniformly placed along each waveguide, and the position of the *m*-th PA on the *n*-th waveguide is given by the following formula, which serves as the reference configuration for the subsequent PA position optimization process:

(4) $$x_{nm}=\frac{\left(m-1\right)L_{\mathrm{wg}}}{M-1}.$$

IM is performed at the waveguide level in the proposed system, where only a subset of the total *N* waveguides is activated in accordance with the index bits carried by the transmitted signal. Specifically, when the *n*-th waveguide (with n∈{1,2,…,N}) is selected via the index bits, all *M* PAs deployed on this *n*-th waveguide radiate simultaneously to transmit the modulation symbols, while the unselected waveguides are not fed by RF chains and their PAs do not radiate modulated signals, thus avoiding intentional co-channel interference and reducing power consumption. The receiver is equipped with $N_{r}$ antennas, which are employed to perform joint detection of the active waveguide indices and the modulation symbols carried by the PAs on the active waveguides, enabling reliable recovery of the transmitted information. In this scheme, the waveguides within the PASS architecture serve as discrete index resources, and this design supports both SM and GSM operating modes to adapt to different transmission requirements. In each transmission time slot, a total of *B* bits is conveyed, where the total number of bits *B* is the sum of the index bits $B_{I}$ and the symbol bits $B_{S}$, which is mathematically expressed as $B=B_{I}+B_{S}.$ In the GSM operating mode, *K* waveguides (with the constraint 1≤K<N) are activated simultaneously. The number of index bits $B_{I}^{\mathrm{GSM}}$ in GSM mode is determined by the number of ways to select *K* waveguides from *N* available waveguides, which is given by

(5) BIGSM=log2NK,

where NK denotes the binomial coefficient representing the combination of *N* waveguides taken *K* at a time, and ⌊·⌋ denotes the floor function to ensure an integer number of index bits. A predefined index mapping function $T_{I}^{\mathrm{GSM}}$ is utilized to map the $B_{I}^{\mathrm{GSM}}$ index bits to the active waveguide set $C^{*}$, which is expressed as

(6) $$C^{*}=\left\{n_{1},\ldots ,n_{K}\right\},$$

where $n_{1},n_{2},\ldots ,n_{K}$ are the indices of the *K* activated waveguides. The mapping follows the conventional lexicographical Gray-coded combinatorial mapping. Unlike conventional GSM schemes that require channel-aware combination selection to avoid highly correlated antenna subsets, the proposed framework first performs global PA position optimization by minimizing the off-diagonal energy of the overall Gram matrix $A^{H}A$. As a result, the equivalent channels associated with all waveguides become nearly orthogonal, yielding uniformly low mutual coherence for every valid *K*-waveguide combination. Therefore, no additional combination screening or adaptive index mapping is required. The number of symbol bits $B_{S}^{\mathrm{GSM}}$ in GSM mode is determined by the number of activated waveguides *K* and the modulation order $M_{\mathrm{mod}}$ of the constellation set M, which is given by $B_{S}^{\mathrm{GSM}}\;=\;K\log_{2}M_{\mathrm{mod}}.$ These $B_{S}^{\mathrm{GSM}}$ symbol bits are mapped to a symbol vector ξ, where ξ belongs to the *K*-th Cartesian product of the constellation set M, i.e., $\xi \in M^{K}.$ The transmit vector s is a sparse vector in the complex space $C^{N\times 1}$, which is constructed by placing the symbol vector ξ at the positions corresponding to the active waveguide set $C^{*}$ in s, and filling all other positions with zeros. This sparse structure of s results in a $\ell_{0}$-norm of *K*, i.e., ${∥s∥}_{0}=K,$ where ${∥s∥}_{0}$ denotes the $\ell_{0}$-norm of s, representing the number of non-zero elements in the vector. It is worth noting that SM is a special case of GSM, which is achieved when the number of activated waveguides K=1.

The PASS channel is characterized by cascaded intra-waveguide and free-space propagation processes, which together govern the signal transmission behavior between the transmitter and receiver. Specifically, the transmitted signal first travels along the dielectric waveguide (intra-waveguide propagation) to each PA and subsequently radiates from the PAs to the receiver through free space (free-space propagation), thus forming the complete PASS channel. This cascaded propagation mechanism directly affects the signal amplitude, phase, and overall transmission reliability, making it essential to model both processes accurately. To precisely characterize the PASS channel, a 3D Cartesian coordinate system is established. Within this coordinate system, the feed point of the *n*-th waveguide (where n∈{1,2,…,N}) is defined as a 3D vector $p_{\mathrm{feed},n}$, mathematically expressed as

(7) $$p_{\mathrm{feed},n}={\left[0,y_{G,n},z_{G,n}\right]}^{T},$$

where $y_{G,n}$ and $z_{G,n}$ denote the *y*-axis and *z*-axis coordinates of the *n*-th waveguide’s feed point, respectively, and the superscript *T* denotes the vector transpose operation. This definition ensures consistent positioning of each waveguide relative to the coordinate system origin. Correspondingly, the position of the *m*-th PA (with m∈{1,2,…,M}) on the *n*-th waveguide is defined as the 3D vector $p_{nm}$, given by

(8) $$p_{nm}={\left[x_{nm},y_{G,n},z_{G,n}\right]}^{T},$$

where $x_{nm}$ is the *x*-axis coordinate of the *m*-th PA on the *n*-th waveguide, which is consistent with the PA position definition provided in the preceding section. The receiver’s position in the 3D Cartesian coordinate system is denoted as r, expressed as

(9) $$r={\left[x_{R},y_{R},z_{R}\right]}^{T},$$

where $x_{R}$, $y_{R}$, and $z_{R}$ represent the *x*-axis, *y*-axis, and *z*-axis coordinates of the receiver, respectively. During intra-waveguide propagation, the signal experiences a phase shift resulting from its travel along the dielectric waveguide. For the *m*-th PA on the *n*-th waveguide, this intra-waveguide phase shift is given by

(10) $$e^{-j\frac{2\pi }{\lambda }n_{\mathrm{eff}}x_{nm}},$$

where *j* is the imaginary unit, λ denotes the carrier wavelength, and $n_{\mathrm{eff}}$ is the effective refractive index of the dielectric waveguide, a key parameter determining the intra-waveguide propagation characteristics. The free-space propagation distance between the *m*-th PA on the *n*-th waveguide and the receiver is the Euclidean distance between the PA position vector $p_{nm}$ and the receiver position vector r, denoted as $r_{nm}$. This distance is calculated as

(11) $$r_{nm}=∥p_{nm}-r∥=\sqrt{{\left(x_{nm}-x_{R}\right)}^{2}+\zeta_{n}^{2}},$$

where ∥·∥ denotes the Euclidean norm, and $\zeta_{n}^{2}$ is a simplified term representing the squared distance between the *n*-th waveguide and the receiver in the *y*-*z* plane. This term is formally defined as

(12) $$\zeta_{n}^{2}={\left(y_{G,n}-y_{R}\right)}^{2}+{\left(z_{G,n}-z_{R}\right)}^{2}.$$

Using the free-space propagation distance $r_{nm}$, the corresponding free-space channel response between the *m*-th PA on the *n*-th waveguide and the receiver is expressed as

(13) $$h_{nm}=\frac{\eta }{r_{nm}}e^{-j\frac{2\pi }{\lambda }r_{nm}},$$

where η is the free-space path loss coefficient, and the exponential term accounts for the phase shift incurred during free-space signal propagation. The overall channel matrix $H\in C^{N_{r}\times NM}$ is constructed by stacking the responses of all PAs. Under the sub-connected architecture, the mapping from RF-chain signals to PA radiation is described by the block-diagonal waveguide radiation matrix

(14) $$G=\mathrm{Blkdiag}(g_{1},\ldots ,g_{N}),$$

where $g_{n}\in C^{M\times 1}$ is the radiation vector of the *n*-th waveguide with

(15) $${\left[g_{n}\right]}_{m}=\sqrt{P_{nm}}e^{-j\frac{2\pi }{\lambda }n_{\mathrm{eff}}x_{nm}}.$$

Let s denote the index-symbol vector generated by IM. The transmitted signal is $x_{\mathrm{ts}}=Gs$, and the received signal is

(16) y=HGs+n=As+n,

where $n∼\mathrm{CN}(0,\sigma^{2}I_{N_{r}})$ and $A=HG\in C^{N_{r}\times N}$ is the equivalent channel matrix. Its (i,n)-th entry is

(17) $$A_{in}=\sum_{m=1}^{M}h_{nm}^{\left(i\right)}\cdot \sqrt{P_{nm}}e^{-j\frac{2\pi }{\lambda }n_{\mathrm{eff}}x_{nm}}.$$

Due to IM, s is a sparse vector satisfying ${∥s∥}_{0}=K$. The joint design objective is to minimize the detection error by optimizing the PA positions for beamforming and the sparse transmit vector

(18) $$\min_{X\in X,\;s\in S}{∥y-A\left(X\right)s∥}_{2}^{2},$$

where $X=\{x_{1},\ldots ,x_{N}\}$ denotes the discrete position set of the PA on each waveguide, X is the feasible domain of PA positions, and S is the feasible domain of the index-symbol vector.

The channel model described above assumes a pure LoS propagation environment, which is typical for PASS deployments in indoor industrial IoT scenarios with clear propagation paths. To characterize more general and practical propagation conditions in which electromagnetic waves experience both direct propagation and passive scattering from surrounding environmental objects, we extend the channel model to the standard Rician fading framework:

(19) $$H=\sqrt{\frac{K_{R}}{K_{R}+1}}H_{\mathrm{LoS}}+\sqrt{\frac{1}{K_{R}+1}}H_{\mathrm{NLoS}}$$

where $K_{R}$ denotes the Rician K-factor, defined as the ratio of the average power of the deterministic LoS component to that of the scattered non-line-of-sight (NLoS) component. The deterministic channel matrix $H_{\mathrm{LoS}}\in C^{N_{r}\times NM}$ represents the direct propagation path between the PASS array and the receiver, whose response is determined by the waveguide radiation characteristics and the free-space propagation described in Equation (17). The random component $H_{\mathrm{NLoS}}\in C^{N_{r}\times NM}$ models the aggregate passive scattering caused by surrounding objects, such as walls, industrial equipment, metallic structures, and other obstacles commonly encountered in indoor deployment environments. Specifically, electromagnetic waves radiated by the pinching antennas undergo multiple reflections, diffraction, and diffuse scattering after interacting with these passive objects, thereby generating numerous indirect propagation paths with random amplitudes, phases, and propagation delays. Under the commonly adopted rich isotropic scattering assumption [26], these scattered paths are regarded as statistically independent. Therefore, according to the central limit theorem, each entry of $H_{\mathrm{NLoS}}$ is modeled as an independent and identically distributed (i.i.d.) circularly symmetric complex Gaussian random variable following CN(0,1). A larger $K_{R}$ corresponds to LoS-dominant propagation with weaker passive scattering, whereas a smaller $K_{R}$ indicates richer scattered multipath propagation.

This statistical modeling approach is adopted for two main reasons. First, under the rich isotropic scattering assumption commonly used for indoor industrial Rician channels, the superposition of a large number of independent scattered paths can be accurately approximated as i.i.d. complex Gaussian random variables according to the central limit theorem. Second, since the primary objective of this work is to evaluate the proposed SA-CDO optimization algorithm and OLS-CC detector rather than environment-specific propagation characteristics, the adopted statistical model effectively isolates algorithmic performance from geometry-dependent factors such as spatial correlation and scatterer distribution. Although geometry-based stochastic channel models (GBSM) can characterize spatial correlation more accurately, they introduce additional environment-dependent parameters that are beyond the scope of this algorithm-oriented study. The integration of GBSM for more realistic deployment analysis is an interesting direction for our future work. For short-range communications where the receiver lies within the radiative near-field region of the PASS array, the far-field plane wave assumption no longer holds. The near-field channel response is modified to account for the spherical wavefront propagation $h_{nm}=\frac{\eta }{r_{nm}^{2}}e^{-j\frac{2\pi }{\lambda }r_{nm}}$, where the path loss scales with $r_{nm}^{-2}$ instead of $r_{nm}^{-1}$ in the far field. It is important to note that the proposed PA position optimization framework and constellation-constrained detection scheme are directly applicable to both far-field and near-field channel models without any modification.

## 3. Index Modulation Transmission Design

Waveguide-based index modulation performs index modulation with waveguides as the basic unit, leveraging the dielectric waveguides in PASS as discrete index resources, where a subset of waveguides is dynamically activated by index bits to convey information. The positions of PAs deployed on the selected active waveguides are jointly optimized to improve the structural properties of the equivalent channel matrix, thereby enhancing the modulation efficiency and detection reliability of the system. To reduce the computational complexity associated with the joint optimization of PA positions and IM detection, a two-stage framework is adopted in the proposed scheme. In the first stage, the PA positions are optimized under practical physical constraints to construct a near-orthogonal equivalent channel matrix. In the second stage, the receiver exploits this structured equivalent channel dictionary to perform joint detection of the active waveguide indices and the modulation symbols carried by the PAs on the active waveguides, ensuring accurate and efficient information recovery.

### 3.1. PA Position Optimization: Simulated Annealing-Based Constrained Discrete Orthogonal Dictionary Construction

The core objective of PA position optimization is to enhance the structural properties of the equivalent channel matrix A(X)=H(X)G(X) by promoting near-orthogonality among its columns. This near-orthogonality is critical for improving the beamforming gain of transmitted signals and enhancing the reliability of sparse recovery at the receiver, which directly determines the overall detection performance of the system. The PA position optimization problem is formally formulated as:

(20) $$\min_{X\in X}{∥I-A^{H}\left(X\right)A\left(X\right)∥}_{F}^{2},$$

where X denotes the feasible set of PA positions defined by discrete physical constraints. Specifically, the feasible set X satisfies:

(21) $$X=\{X|x_{nm}\in \left[0,L_{wg}\right],\;x_{n(m+1)}-x_{nm}\geq \lambda /2\},$$

where $x_{nm}\in \left[0,L_{wg}\right]$ enforces the boundary constraint that each PA must be located within the waveguide length, and $x_{n(m+1)}-x_{nm}\geq \lambda /2$ imposes the minimum spacing constraint to suppress mutual coupling between adjacent PAs. Let $A=[u_{1},\ldots ,u_{N}]$ denote the equivalent channel matrix, where the *n*-th column $u_{n}$ is defined as the Hadamard product of the radiation vector $g_{n}\left(x_{n}\right)$ and the channel response vector $h_{n}\left(x_{n}\right)$ of the *n*-th waveguide, i.e., $u_{n}=g_{n}\left(x_{n}\right)⊙h_{n}\left(x_{n}\right)$. This vector $u_{n}$ represents the equivalent radiation-channel response vector of the *n*-th waveguide, integrating the effects of intra-waveguide propagation, PA radiation, and free-space propagation.

A key feature of PASS is the block-diagonal structure of the radiation matrix G. Consequently, each equivalent dictionary atom $u_{n}$ depends only on the PA positions of its corresponding *n*-th waveguide and is independent of other waveguides. This property decomposes the high-dimensional global optimization problem into multiple low-dimensional single-waveguide subproblems, significantly reducing computational complexity. At the *k*-th iteration, with all waveguides fixed except the *n*-th, the residual matrix is defined as:

(22) $$R_{n}^{\left(k\right)}=I-\sum_{i\neq n}u_{i}^{\left(k\right)}u_{i}^{(k)H}.$$

The local optimization problem for the *n*-th waveguide can then be formulated as:

(23) $$\min_{x_{n}\in X_{n}}{∥R_{n}^{\left(k\right)}-u_{n}\left(x_{n}\right)u_{n}^{H}\left(x_{n}\right)∥}_{F}^{2},$$

where $X_{n}$ denotes the discrete feasible set of PA positions for the *n*-th waveguide. Using the fundamental property of the Frobenius norm:

(24) $${∥A∥}_{F}^{2}=\mathrm{Tr}\;\left(A^{H}A\right),$$

the objective function can be expanded as:

(25) $$\begin{array}{cc} {∥R_{n}^{\left(k\right)}-u_{n}u_{n}^{H}∥}_{F}^{2}\; & =\mathrm{Tr}\;\left[{\left(R_{n}^{\left(k\right)}-u_{n}u_{n}^{H}\right)}^{H}\left(R_{n}^{\left(k\right)}-u_{n}u_{n}^{H}\right)\right]\\ \; & \;=\mathrm{Tr}\;\left(R_{n}^{\left(k\right)}R_{n}^{\left(k\right)}\right)-2\;\mathrm{Tr}\;\left(R_{n}^{\left(k\right)}u_{n}u_{n}^{H}\right)+\mathrm{Tr}\;\left(u_{n}u_{n}^{H}u_{n}u_{n}^{H}\right). \end{array}$$

Note that $\mathrm{Tr}\;\left(R_{n}^{\left(k\right)}R_{n}^{\left(k\right)}\right)$ is independent of $x_{n}$ and can be regarded as a constant term. Since the equivalent atom $u_{n}$ is approximately power-normalized, $\mathrm{Tr}\;\left(u_{n}u_{n}^{H}u_{n}u_{n}^{H}\right)$ can also be treated as a constant. Accordingly, the original minimization problem is equivalently transformed into:

(26) $$\min_{x_{n}\in X_{n}}{∥R_{n}^{\left(k\right)}-u_{n}\left(x_{n}\right)u_{n}^{H}\left(x_{n}\right)∥}_{F}^{2}=\max_{x_{n}\in X_{n}}\mathrm{Tr}\left(u_{n}^{H}\left(x_{n}\right)R_{n}^{\left(k\right)}u_{n}\left(x_{n}\right)\right).$$

For ∀n, by omitting unnecessary superscripts and subscripts for notational simplicity, the local objective function simplifies to:

(27) $$\max\mathrm{Tr}\;\left(u^{H}\left(x\right)Ru\left(x\right)\right).$$

By exploiting the cyclic permutation property of the trace operator and the rank-1 structure of $u\left(x\right)u^{H}\left(x\right)$, the objective function can be further reduced to the following quadratic form:

(28) $$\mathrm{Tr}\;\left(u^{H}\left(x\right)Ru\left(x\right)\right)={\left(g(x)⊙h(x)\right)}^{H}R\left(g(x)⊙h(x)\right).$$

The objective function depends on the PA position vector x through the phase responses embedded in g(x) and h(x), resulting in a high-dimensional nonconvex optimization problem defined over the discrete set $X_{n}$. This problem exhibits numerous local optima, and traditional gradient-based methods are susceptible to numerical errors and often fail to converge to the global optimum. This challenge is exacerbated when the number of PAs per waveguide M≥8, as the nonconvexity of the objective function becomes significantly more pronounced.

To address this limitation, this paper proposes a Simulated Annealing-based Constrained Discrete Optimization (SA-CDO) algorithm. SA is a probabilistic metaheuristic inspired by the annealing process in metallurgy. It accepts worse solutions with a temperature-controlled probability, enabling effective escape from local optima and yielding stable convergence to high-quality near-optimal solutions under a properly designed cooling schedule. Furthermore, SA naturally adapts to discrete constrained problems without requiring gradient information, making it particularly suitable for the PA position optimization problem with strict physical constraints.

**Figure 2:**
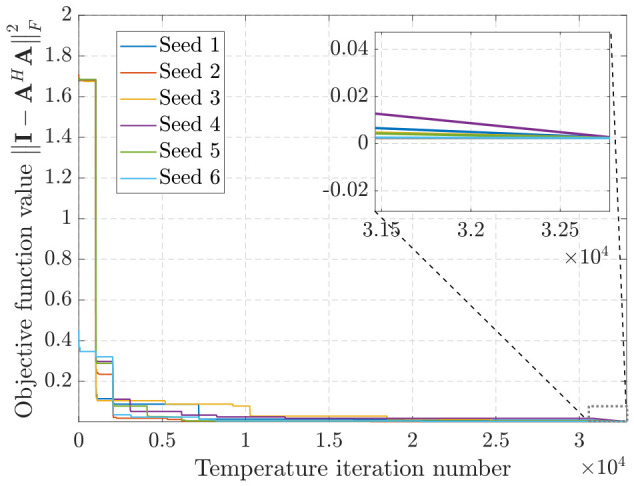
Convergence trajectories of the proposed SA-CDO algorithm under six independent random seeds. The inset shows a zoomed-in view of the final convergence stage. System parameters: $L_{\max}=800$, α=0.98.

The core idea of SA-CDO is to start from the initial uniform PA configuration, iteratively generate constraint-satisfying neighbor solutions, and accept or reject them according to the Metropolis criterion. To mitigate the risk of premature convergence associated with static cooling schedules, a slow cooling rate (α=0.98) together with sufficient equilibrium iterations per temperature level ($L_{\max}=800$) is adopted, allowing extensive neighborhood exploration before each temperature reduction. In addition, the proposed sequential alternating optimization framework repeatedly optimizes different waveguides across multiple outer iterations, effectively introducing multiple search trajectories and providing additional opportunities to escape local minima. Furthermore, a temperature-synchronized neighborhood step decay is employed: larger perturbations are maintained during high-temperature stages to enhance global exploration, while smaller perturbations are gradually adopted during low-temperature stages to improve local refinement accuracy. As the temperature decreases, the probability of accepting worse solutions gradually diminishes, enabling a smooth transition from global exploration to local exploitation. Figure 2 illustrates the convergence trajectories of the proposed SA-CDO algorithm under six independent random seeds, with a local zoom-in view highlighting the final convergence state. Empirically, the maximum relative deviation of the final objective values across repeated independent runs is approximately 0.64%, indicating that the proposed SA-CDO algorithm exhibits stable convergence behavior and effectively avoids severe local-minimum trapping under the considered parameter settings. The algorithm converges to a high-quality PA position configuration that maximizes the objective function. The complete optimization procedure is summarized in Algorithm 1.
**Algorithm 1** Simulated Annealing-Based Constrained Discrete PA Position Optimization**Require:** Residual matrix $R_{n}$, initial PA position vector $x_{n}^{\left(0\right)}\in R^{M\times 1}$ (uniform distribution), initial temperature $T_{0}$, cooling rate α, minimum temperature $T_{\min}$, neighborhood perturbation step δ, maximum iterations per temperature $L_{\max}$**Ensure:** Optimized discrete PA position vector $x_{n}^{★}\in X_{n}$  1:Initialize: $T\leftarrow T_{0}$, $x_{\mathrm{curr}}\leftarrow x_{n}^{\left(0\right)}$, $x_{\mathrm{best}}\leftarrow x_{n}^{\left(0\right)}$  2:Compute initial objective value: $J_{\mathrm{curr}}=u^{H}\left(x_{\mathrm{curr}}\right)R_{n}u\left(x_{\mathrm{curr}}\right)$, $J_{\mathrm{best}}\leftarrow J_{\mathrm{curr}}$  3:**while** $T>T_{\min}$ **do**  4:   **for** l=1 **to** $L_{\max}$ **do**  5:     Generate constraint-satisfying neighbor solution $x_{\mathrm{neigh}}$ from $x_{\mathrm{curr}}$:  6:        Randomly select one PA index m∈{1,2,…,M}  7:        Perturb its position: $x_{\mathrm{neigh}}\left[m\right]=x_{\mathrm{curr}}\left[m\right]+\mathrm{rand}\left(\right)\cdot \delta -\delta /2$  8:        Enforce boundary constraint: $x_{\mathrm{neigh}}\left[m\right]=\max\left(0,\min\left(L_{\mathrm{wg}},x_{\mathrm{neigh}}\left[m\right]\right)\right)$  9:        Enforce minimum spacing constraint:10:           - If m>1 and $x_{\mathrm{neigh}}\left[m\right]-x_{\mathrm{neigh}}\left[m-1\right]<\lambda /2$,           set $x_{\mathrm{neigh}}\left[m\right]=x_{\mathrm{neigh}}\left[m-1\right]+\lambda /2$11:           - If m<M and $x_{\mathrm{neigh}}\left[m+1\right]-x_{\mathrm{neigh}}\left[m\right]<\lambda /2$,           set $x_{\mathrm{neigh}}\left[m+1\right]=x_{\mathrm{neigh}}\left[m\right]+\lambda /2$12:     Compute neighbor objective value: $J_{\mathrm{neigh}}=u^{H}\left(x_{\mathrm{neigh}}\right)R_{n}u\left(x_{\mathrm{neigh}}\right)$13:     Calculate acceptance probability: $p=\exp\left(\frac{J_{\mathrm{neigh}}-J_{\mathrm{curr}}}{T}\right)$14:     **if** $J_{\mathrm{neigh}}>J_{\mathrm{curr}}$ **or** rand()<p **then**15:        $x_{\mathrm{curr}}\leftarrow x_{\mathrm{neigh}}$, $J_{\mathrm{curr}}\leftarrow J_{\mathrm{neigh}}$16:     **end if**17:     **if** $J_{\mathrm{curr}}>J_{\mathrm{best}}$ **then**18:        $x_{\mathrm{best}}\leftarrow x_{\mathrm{curr}}$, $J_{\mathrm{best}}\leftarrow J_{\mathrm{curr}}$19:     **end if**20:   **end for**21:   Cool down the temperature: T←α·T22:**end while**23:**return** $x_{n}^{★}=x_{\mathrm{best}}$

#### 3.1.1. Algorithm Complexity Analysis

The time complexity of the SA-CDO algorithm can be hierarchically decomposed into three layers: global alternating optimization, single-waveguide simulated annealing iteration, and per-iteration objective function evaluation. To avoid high-dimensional matrix operations, a key mathematical simplification is first introduced: leveraging the definition of the residual matrix $R_{n}=I-\sum_{i\neq n}u_{i}u_{i}^{H}$, the objective function can be expanded as:

(29) $$J=u^{H}R_{n}u={∥u∥}_{2}^{2}-\sum_{i\neq n}{\left|u^{H}u_{i}\right|}^{2},$$

This expression reduces the original $O(N_{r}^{2})$ matrix-vector multiplication to $O(N_{r}\left(M+N\right))$ vector inner product operations, where *M* is the number of PAs per waveguide, *N* is the total number of waveguides, and $N_{r}$ is the number of receive antennas.

The complexity of a single objective function evaluation is dominated by two steps: (1) generating the equivalent channel atom u(x) by superposing the channel responses of *M* PAs, with complexity $O(MN_{r})$; (2) computing the cross-correlation terms between u and the remaining N−1 waveguide atoms, with complexity $O(NN_{r})$. Thus, the overall complexity of a single objective function evaluation is $O(N_{r}\left(M+N\right))$. For single-waveguide SA-CDO optimization, let $T_{\mathrm{total}}$ denote the total number of temperature iterations and $L_{\max}$ denote the maximum number of neighborhood search iterations per temperature. The complexity of operations in a single iteration, such as neighbor solution generation, constraint enforcement, and Metropolis acceptance judgment, is negligible compared to the objective function evaluation. Therefore, the complexity of optimizing a single waveguide is $O(T_{\mathrm{total}}\cdot L_{\max}\cdot N_{r}\left(M+N\right))$. Benefiting from the block-diagonal structure of the PASS radiation matrix, the global optimization adopts a sequential alternating framework: fix all other waveguides, optimize the PA positions of each waveguide sequentially, and repeat this process for $I_{\mathrm{alt}}$ iterations. The overall time complexity of the global SA-CDO algorithm is:

(30) $$O\left(I_{\mathrm{alt}}\cdot N\cdot T_{\mathrm{total}}\cdot L_{\max}\cdot N_{r}\left(M+N\right)\right).$$

#### 3.1.2. Robustness Analysis Under Rician Fading

The above analysis verifies the effectiveness of the proposed SA-CDO algorithm under pure LoS conditions, but evaluating its robustness in practical scattering environments is equally crucial. Extending the analysis to the Rician fading channel model introduced in Section 2, the equivalent channel matrix under Rician fading can be expressed as:

(31) $$A=\sqrt{\frac{K_{R}}{K_{R}+1}}A_{\mathrm{LoS}}\left(X\right)+\sqrt{\frac{1}{K_{R}+1}}A_{\mathrm{NLoS}}$$

where $A_{\mathrm{LoS}}\left(X\right)=H_{\mathrm{LoS}}G\left(X\right)$ is the optimized LoS equivalent channel matrix, and $A_{\mathrm{NLoS}}=H_{\mathrm{NLoS}}G\left(X\right)$ is the random NLoS equivalent channel matrix whose entries follow an independent and identically distributed circularly symmetric complex Gaussian distribution CN(0,1). The corresponding Gram matrix is:

(32) $$\begin{array}{cc} G_{A} & =A^{H}A=\frac{K_{R}}{K_{R}+1}A_{\mathrm{LoS}}^{H}A_{\mathrm{LoS}}\\ & +\frac{\sqrt{K_{R}}}{K_{R}+1}\left(A_{\mathrm{LoS}}^{H}A_{\mathrm{NLoS}}+A_{\mathrm{NLoS}}^{H}A_{\mathrm{LoS}}\right)\\ & +\frac{1}{K_{R}+1}A_{\mathrm{NLoS}}^{H}A_{\mathrm{NLoS}} \end{array}$$

Since SA-CDO performs global optimization on the above deterministic objective function and obtains a superior global solution, the optimized LoS equivalent channel matrix $A_{\mathrm{LoS}}$ satisfies $A_{\mathrm{LoS}}^{H}A_{\mathrm{LoS}}\approx I$ with higher accuracy. Given that the cross terms are zero-mean random variables, the expected value of the Gram matrix satisfies:

(33) $$E\left[G_{A}\right]\approx \frac{K_{R}}{K_{R}+1}I+\frac{1}{K_{R}+1}I=I$$

This theoretical result demonstrates that PA positions optimized by SA-CDO maintain the near-orthogonality of the equivalent channel matrix even in the presence of NLoS scattering components. Furthermore, due to the improved orthogonality achieved by SA-CDO, performance degradation caused by scattering is further mitigated. This robustness property makes the proposed scheme suitable for a wider range of practical deployment scenarios beyond ideal LoS environments.

Remarkably, the above analysis reveals that the deterministic optimization problem formulated in Equation (21) is actually the optimal solution to a more general robust stochastic optimization problem:

(34) $$\min_{X\in X}E\left[{∥I-A^{H}\left(X\right)A\left(X\right)∥}_{F}^{2}\right],$$

where the expectation is taken over all random NLoS channel realizations. This equivalence holds because NLoS components are zero-mean and statistically independent of the deterministic LoS component and thus do not affect the optimal PA position configuration. This is a key theoretical insight: the proposed SA-CDO algorithm is inherently robust to NLoS scattering by design, not as an afterthought. It helps achieve improved average performance across all considered channel realizations, which is precisely what is required for reliable communications in practical 6G IoT environments.

To quantitatively characterize the structural impact of SA-CDO optimization on the equivalent channel, the Gram matrix of A is defined as $G_{A}=A^{H}A$. The diagonal entries ${\left[G_{A}\right]}_{ii}$ represent the self-correlation of the *i*-th waveguide channel and reflect its effective radiation energy, while the off-diagonal entries ${\left[G_{A}\right]}_{ij}$ (i≠j) characterize inter-waveguide correlation and coupling interference.

**Figure 3:**
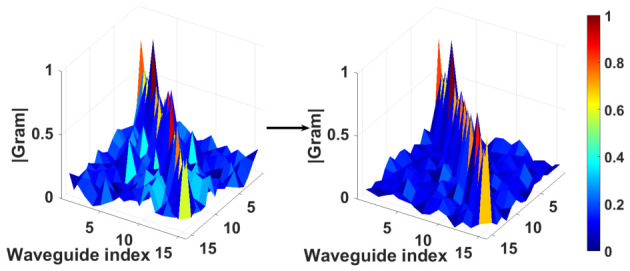
Heatmap comparison of the equivalent channel Gram matrix $G_{A}=A^{H}A$ before and after SA-CDO PA position optimization under Rician fading ($K_{R}=50$). System parameters: N=16, M=8, $L_{\mathrm{wg}}=20\;m$, $n_{\mathrm{eff}}=1.5$, λ=0.05m, $d_{\mathrm{wg}}=0.3\;m$, and $N_{r}=24$.

Figure 3 compares the heatmaps of $G_{A}$ before and after SA-CDO optimization under Rician fading. With the initial uniform PA deployment, the off-diagonal entries exhibit relatively large magnitudes, i.e., $|{\left[G_{A}\right]}_{ij}|\gg 0$ for i≠j, indicating strong inter-waveguide correlation that increases the difficulty of index detection and sparse recovery. After applying the proposed SA-CDO algorithm, the optimized PA position set $X^{★}$ yields $|{\left[G_{A}\right]}_{ij}|\ll {\left[G_{A}\right]}_{ii}$ for all i≠j, demonstrating that the equivalent channel matrix exhibits a pronounced near-orthogonal structure. Even in the presence of NLoS scattering components, the magnitudes of the off-diagonal entries remain significantly smaller than those of the diagonal entries, further verifying the structural robustness of the proposed SA-CDO optimization under Rician fading. This near-orthogonal property effectively suppresses inter-waveguide interference and improves the distinguishability of active waveguide indices, thereby providing stable and reliable channel conditions for constellation-constrained sparse detection based on the optimized equivalent channel matrix $A^{★}=A\left(X^{★}\right)$.

### 3.2. Sparse Symbol Recovery: Orthogonal Least Squares-Based Constellation-Constrained Detection

Upon completion of the PA position optimization, the optimized equivalent channel matrix $A^{*}=A\left(X^{*}\right)$ is treated as accurately known and fixed at the receiver within one channel coherence interval. The core task of the receiver is to reconstruct the sparse index-symbol vector s from the noisy received observation, achieving joint detection of active waveguide indices and corresponding modulation symbols. The received signal model is given by:

(35) $$y=A^{*}s+n,$$

where the sparse index-symbol vector s is subject to two fundamental constraints: first, its $\ell_{0}$-norm satisfies ${∥s∥}_{0}=K$ with K<N, indicating that exactly *K* waveguides are activated; second, all nonzero entries of s are drawn from a predefined modulation constellation set M.

Accordingly, the sparse symbol recovery problem at the receiver can be mathematically formulated as the following constrained least-squares optimization problem:

(36) $$\begin{array}{cc} \min_{s} & {∥y-A^{*}s∥}_{2}^{2}\\ s.t. & {∥s∥}_{0}=K,\\ & s_{n}\in M\cup \left\{0\right\},\;\forall n\in \left\{1,2,\ldots ,N\right\}, \end{array}$$

where $s={\left[s_{1},s_{2},\ldots ,s_{N}\right]}^{T}\in C^{N\times 1}$ is the sparse transmit vector. Specifically, $s_{n}=0$ indicates that the *n*-th waveguide is inactive and does not transmit any signal, whereas $s_{n}\in M$ corresponds to an active waveguide that carries a modulation symbol from the constellation set M.

To efficiently solve this problem, this paper proposes an Orthogonal Least Squares-based Constellation-Constrained (OLS-CC) detector. Unlike exhaustive maximum likelihood (ML) detection, the proposed detector decouples sparse support recovery and constellation projection, significantly reducing computational complexity while maintaining reliable detection performance. In particular, OLS-CC exhibits natural compatibility with the near-orthogonal equivalent channel constructed by the aforementioned SA-CDO algorithm: the orthogonalization operation significantly reduces the influence of previously selected atoms on the residual, mitigating repeated selection and error propagation, and achieves performance close to the optimal ML detection under near-orthogonal channel conditions.

The core idea of the OLS algorithm is to iteratively select the equivalent channel atom (corresponding to the active waveguide index) with the maximum correlation to the current residual. After each selection, QR orthogonalization is immediately performed on the set of selected atoms, making the updated residual completely orthogonal to all selected atoms, thus effectively preventing subsequent iterations from repeatedly selecting already activated waveguides. After completing *K* iterations to obtain the complete support set, the soft symbols obtained by least-squares estimation are projected onto the nearest constellation points to satisfy the modulation constraint. The complete detection procedure is summarized in Algorithm 2.

#### 3.2.1. Algorithm Complexity Analysis and Performance Advantages

Compared with exhaustive ML detection, the OLS-CC detector avoids combinatorial index-symbol search by decoupling sparse support recovery and symbol refinement and achieves reliable sparse recovery by leveraging the near-orthogonal channel structure constructed by SA-CDO. For conventional ML detection, all possible active waveguide combinations and modulation symbol combinations must be exhaustively searched, resulting in a time complexity of: ONK|M|KNrK. This complexity grows exponentially with the sparsity level *K*, making it practically infeasible in medium and large-scale systems.

In contrast, the time complexity of the OLS-CC detector consists of two components. In the support selection stage, the inner product of *N* $N_{r}$-dimensional vectors needs to be calculated in each iteration, and the total complexity of *K* iterations is $O(KN_{r}N)$. In the orthogonalization and least-squares estimation stage, incremental QR decomposition is performed on the $t\times N_{r}$ matrix in each iteration, and the total complexity of *K* iterations is $O(K^{2}N_{r})$. Therefore, the overall time complexity of the OLS-CC detector is

(37) $$O(KN_{r}N+K^{2}N_{r}),$$

which falls into the polynomial-time complexity category and exhibits excellent engineering implementability under the system parameter configuration adopted in this paper.
**Algorithm 2** Orthogonal Least Squares Based Constellation-Constrained Sparse Symbol Detection**Require:** Received signal y, optimized equivalent channel matrix $A^{*}$, sparsity level *K*, modulation constellation set M**Ensure:** Recovered sparse index-symbol vector $\hat{s}$  1:Initialize residual $r^{\left(0\right)}=y$, support set $C^{\left(0\right)}=Ø$, selected atom matrix $A_{C}^{\left(0\right)}=\left[\right]$, iteration index t=1  2:**while** t≤K **do**  3:   Compute correlation coefficients between residual and all candidate atoms:   $\rho_{n}=\left|a_{n}^{H}r^{\left(t-1\right)}\right|,\;\forall n\notin C^{\left(t-1\right)}$  4:   Select the index with maximum correlation:   $n_{t}=\arg\max_{n\notin C^{\left(t-1\right)}}\rho_{n}$  5:   Update support set: $C^{\left(t\right)}=C^{\left(t-1\right)}\cup \left\{n_{t}\right\}$  6:   Update selected atom matrix: $A_{C}^{\left(t\right)}=\left[A_{C}^{\left(t-1\right)}\;a_{n_{t}}\right]$  7:   Perform incremental QR update: $\left[Q^{\left(t\right)},R^{\left(t\right)}\right]=\mathrm{UpdateQR}\left(Q^{\left(t-1\right)},R^{\left(t-1\right)},a_{n_{t}}\right)$  8:   Compute least squares estimate via orthogonal projection: ${\hat{\xi }}^{\left(t\right)}={\left(R^{\left(t\right)}\right)}^{-1}{\left(Q^{\left(t\right)}\right)}^{H}y$  9:   Update residual (orthogonal to all selected atoms): $r^{\left(t\right)}=y-A_{C}^{\left(t\right)}{\hat{\xi }}^{\left(t\right)}$10:   t=t+111:**end while**12:Project estimated soft symbols onto the nearest constellation points:${\hat{\zeta }}_{k}=\arg\min_{\xi \in M}{\left|{\hat{\xi }}_{k}^{\left(K\right)}-\xi \right|}^{2},\;\forall k=1,2,\ldots ,K$13:Construct recovered sparse vector: ${\hat{s}}_{C^{\left(K\right)}}=\hat{\zeta },\;{\hat{s}}_{\bar{C^{\left(K\right)}}}=0$14:**return** $\hat{s}$

To quantitatively demonstrate the computational advantage of the proposed framework, Table 1 summarizes the computational complexity of representative transceiver schemes. The transmitter-side complexity corresponds to the one-time PA position optimization executed in the offline configuration phase, whereas the receiver-side complexity corresponds to the online detection performed for each transmission slot.

As shown in Table 1, exhaustive-search-based PA optimization exhibits exponential complexity with respect to the PA configuration space, making it computationally prohibitive even for moderate system dimensions. Similarly, the conventional ML detector requires exhaustive enumeration over all possible active waveguide combinations and modulation symbols, resulting in exponential complexity with respect to the sparsity level and modulation order. Consequently, exhaustive-search-based transceiver designs are impractical for medium- and large-scale PASS systems. In contrast, the proposed SA-CDO optimization algorithm achieves polynomial-time complexity by sequentially optimizing the PA positions under physical constraints, thereby avoiding exhaustive exploration of the entire configuration space. On the receiver side, both the conventional OLS detector and the proposed OLS-CC detector exhibit polynomial complexity dominated by $O(KN_{r}N+K^{2}N_{r})$. The additional constellation projection step in OLS-CC introduces only O(K|M|) extra computations, which are negligible compared with the dominant QR updating and correlation calculation operations. It is worth noting that the transmitter-side PA optimization is executed only once during the offline system configuration stage, whereas the receiver-side detection is performed online for every transmission slot. Therefore, the computational burden during real-time operation is dominated by the polynomial-complexity OLS-CC detector. Overall, the proposed transceiver framework effectively reduces the online detection complexity from exponential to polynomial order while maintaining superior detection performance, making it well-suited for practical medium- and large-scale PASS-based waveguide index modulation systems and low-latency 6G IoT scenarios.

It is well recognized in numerical linear algebra that incremental QR updating may gradually lose orthogonality when the selected atom matrix becomes severely ill-conditioned [27]. In the proposed OLS-CC detector, however, this risk is substantially alleviated owing to both the pre-optimized channel dictionary constructed by the SA-CDO algorithm and the inherent atom selection mechanism of OLS. Specifically, the equivalent channel dictionary used for sparse detection is not randomly generated, but is explicitly optimized by the SA-CDO algorithm. During the PA position optimization stage, SA-CDO directly minimizes the off-diagonal energy of the channel Gram matrix, thereby significantly reducing the mutual coherence among waveguide channel atoms. Compared with conventional fixed-PA deployment, the optimized dictionary exhibits substantially weaker linear dependence among candidate atoms. Consequently, the atom submatrix selected during the OLS iterations is generally much better conditioned, which fundamentally reduces the likelihood of numerical orthogonality loss during incremental QR updating. Furthermore, the atom selection strategy of OLS provides an additional level of numerical robustness. Unlike Orthogonal Matching Pursuit (OMP), which selects atoms solely according to their instantaneous correlation with the residual, OLS performs an orthogonal projection onto the current support subspace at each iteration and chooses the candidate atom that yields the largest reduction in the residual norm. This selection criterion effectively avoids repeatedly selecting highly correlated atoms, thereby further improving the numerical stability of the subsequent QR updating process. For extremely ill-conditioned propagation scenarios requiring stronger numerical robustness, modified Gram–Schmidt orthogonalization with reorthogonalization or Householder QR decomposition can be employed as alternative implementations. These approaches provide superior numerical stability at the cost of only a modest increase in computational complexity, while remaining fully compatible with the proposed detection framework.

#### 3.2.2. Detection Performance Under Rician Fading Channels

In practical propagation environments, the received signal is affected by both deterministic LoS components and random scattering components. According to the Rician channel model introduced in Section 2, the optimized equivalent channel matrix can be expressed as:

(38) $$A^{*}=\sqrt{\frac{K_{R}}{K_{R}+1}}A_{\mathrm{LoS}}^{*}+\sqrt{\frac{1}{K_{R}+1}}A_{\mathrm{NLoS}},$$

where $A_{\mathrm{LoS}}^{*}$ denotes the LoS equivalent channel matrix optimized by the SA-CDO algorithm, and $A_{\mathrm{NLoS}}$ represents the random scattering component whose entries follow an independent and identically distributed circularly symmetric complex Gaussian distribution CN(0,1). The sparse recovery performance of OLS-CC is closely related to the mutual coherence of the equivalent channel matrix, which is defined as the maximum absolute inner product between any two distinct atoms in the dictionary:

(39) $$\mu \left(A\right)=\max_{i\neq j}\frac{\left|a_{i}^{H}a_{j}\right|}{∥a_{i}{∥}_{2}{∥a_{j}∥}_{2}}.$$

A smaller mutual coherence indicates weaker correlation between atoms and generally leads to more reliable sparse support recovery. Since the proposed SA-CDO algorithm explicitly minimizes the off-diagonal energy of the Gram matrix, the optimized LoS component satisfies:

(40) $$\mu \left(A_{\mathrm{LoS}}^{*}\right)\ll \mu \left(A_{\mathrm{uniform}}\right),$$

where $A_{\mathrm{uniform}}$ denotes the equivalent channel matrix generated by the conventional uniform PA deployment scheme. Under Rician fading conditions, the NLoS component introduces random perturbations into the equivalent channel matrix. However, when the Rician factor $K_{R}$ is moderate or large (a typical value of $K_{R}\geq 5$ for industrial Internet of Things scenarios), the optimized LoS component remains dominant. Consequently, the mutual coherence of the overall equivalent channel matrix can be approximated as:

(41) $$\mu \left(A^{*}\right)\approx \mu \left(A_{\mathrm{LoS}}^{*}\right)+\Delta_{\mathrm{NLoS}},$$

where $\Delta_{\mathrm{NLoS}}$ represents the coherence perturbation induced by scattering. Since the optimized LoS component already exhibits strong near-orthogonality, the overall mutual coherence remains significantly lower than that of conventional PASS configurations even in the presence of scattering perturbations.

Under this condition, the correlation-based atom selection mechanism of OLS-CC can still identify the correct active waveguide indices with high probability. Furthermore, the orthogonal projection step of OLS-CC can completely eliminate the contribution of previously selected atoms from the residual, thereby suppressing error propagation caused by channel perturbations. Therefore, the proposed OLS-CC detector maintains reliable sparse support recovery capability even in the presence of multipath scattering. From a geometric perspective, the SA-CDO algorithm increases the angular separation between equivalent channel atoms, while the OLS-CC algorithm further amplifies this separation effect through iterative orthogonal projections. The synergistic effect of these two mechanisms significantly improves the distinguishability of active waveguide indices under Rician fading. Consequently, the proposed receiver maintains a low bit error rate over a wide range of Rician factors and signal-to-noise ratio regimes, demonstrating strong robustness against practical channel impairments.

### 3.3. Asymptotic BER Analysis and Diversity Performance

To provide theoretical insight into the error performance of the proposed PASS-based waveguide index modulation scheme, this subsection derives the asymptotic BER in the high-SNR regime based on the pairwise error probability (PEP) framework. The resulting BER upper bound is further employed to characterize the achievable diversity order and array gain of the proposed transceiver architecture. The optimal ML detector is adopted as the theoretical benchmark, and the derived performance bound serves as a reference for evaluating the proposed OLS-CC detector. Consider two distinct index-symbol vectors $s_{i}$ and $s_{j}$. Under the additive white Gaussian noise (AWGN) channel, the pairwise error probability of erroneously deciding $s_{j}$ when $s_{i}$ is transmitted is given by

(42) $$P\left(s_{i}\to s_{j}\right)=Q\left(\frac{{∥A(s_{i}-s_{j})∥}_{2}^{2}}{2\sigma^{2}}\right),$$

where Q(·) denotes the Gaussian *Q*-function, A is the equivalent channel matrix, and $\sigma^{2}$ represents the noise variance. In the high-SNR regime, the Gaussian *Q*-function can be upper bounded by $Q\left(x\right)\leq \frac{1}{2}e^{-x^{2}/2}.$ For the proposed system with *K* activated waveguides, the equivalent channel atoms (i.e., the columns of A) become approximately orthogonal after the SA-CDO optimization. Under this near-orthogonal condition, the minimum squared Euclidean distance between any two codewords can be approximated as

(43) $$d_{\min}^{2}=\min_{i\neq j}{∥A(s_{i}-s_{j})∥}_{2}^{2}\approx K\cdot d_{\mathrm{symbol}}^{2}{∥u_{n}∥}_{2}^{2},$$

where $d_{\mathrm{symbol}}^{2}$ denotes the minimum Euclidean distance of the modulation constellation, and ${∥u_{n}∥}_{2}^{2}$ represents the equivalent channel gain associated with the *n*-th waveguide. Based on the union bound, the average BER of the proposed system can be asymptotically upper bounded at high SNR as

(44) $$P_{b}\leq \frac{N_{c}}{2B}\exp\left(-\frac{K\cdot d_{\mathrm{symbol}}^{2}{∥u_{n}∥}_{2}^{2}}{4\sigma^{2}}\right),$$

where $N_{c}$ denotes the number of nearest-neighbor codewords and *B* is the total number of information bits transmitted per time slot.

Several important observations can be drawn from the above asymptotic expression. Under the considered single-RF-chain-per-waveguide architecture, the proposed waveguide index modulation scheme achieves a diversity order of one, which is consistent with the fundamental diversity characteristic of conventional GSM. When multiple receive antennas are employed, additional receive diversity can be exploited, and the achievable diversity order increases proportionally with the number of receive antennas. Meanwhile, the proposed SA-CDO algorithm further improves the system performance by optimizing the PA positions to enhance the equivalent channel gain $∥u_{n}{∥}_{2}^{2}$, thereby providing additional array gain. Furthermore, the near-orthogonal equivalent channel dictionary constructed by SA-CDO significantly reduces the mutual coherence among waveguide channels, effectively suppresses inter-waveguide interference, and enlarges the minimum Euclidean distance between codewords. Therefore, under the considered single-RF-chain architecture, the proposed PASS-based waveguide index modulation scheme preserves the same diversity order as conventional GSM while achieving substantial coding gain, or equivalently, array gain, through SA-CDO-based channel orthogonalization. Consequently, the proposed framework significantly improves the BER performance without increasing the online detection complexity. This theoretical conclusion is fully consistent with the simulation results presented in Section 4.

## 4. Simulation Results

This section comprehensively evaluates the proposed waveguide-based index modulation scheme under both pure LoS and Rician fading channels through Monte Carlo simulations. All numerical results are averaged over 30,000 independent channel realizations. Moreover, for each SNR point, the simulation continues until at least 200 bit errors are collected before the BER is evaluated. This stopping criterion is widely adopted in Monte Carlo simulations to reduce statistical fluctuations and improve the reliability of BER estimation, particularly in the low-error-rate region. All compared schemes are simulated using identical random seeds and channel realizations to ensure a statistically fair comparison. Unless otherwise specified, the default system parameters are set as follows: the total number of waveguides is N=16, the carrier wavelength is λ=0.05m, the effective refractive index of the dielectric waveguide is $n_{\mathrm{eff}}=1.5$, the inter-waveguide spacing is $d_{\mathrm{wg}}=0.2\;m$, the number of receive antennas is $N_{r}=16$, and the receiver distance is $x_{R}=25\;m$. For the Rician fading scenario, the default Rician factor is set to $K_{R}=50$, corresponding to a typical industrial indoor multipath environment, while the pure LoS scenario corresponds to the limiting case $K_{R}\to \infty$. The number of PAs deployed on each waveguide is selected from M∈{1,4,8,10,12} to cover different deployment scales. Quadrature phase shift keying (QPSK) modulation is adopted, and the BER is used as the primary performance metric. The transmitter employs a sub-connected architecture, where the equivalent channel matrix has dimension $N_{r}\times N$, and the PA positions are optimized via the proposed SA-CDO algorithm (Algorithm 1).

For each Monte Carlo realization, the deterministic LoS channel matrix is first generated according to the geometric configuration described in Section 2. Specifically, the propagation distance between each PA and each receive antenna is calculated using Equation (11), and the deterministic LoS channel matrix $H_{\mathrm{LoS}}$ is subsequently constructed according to Equation (17), where both the free-space propagation phase and the intra-waveguide phase shift are incorporated. The NLoS component is then generated as an independent and identically distributed circularly symmetric complex Gaussian random matrix whose entries follow CN(0,1), representing the aggregate passive scattering introduced by surrounding objects in practical industrial environments. The overall propagation channel is synthesized according to Equation (19) by combining the deterministic LoS and stochastic NLoS components under the prescribed Rician factor $K_{R}$, whereas only the deterministic component is retained for the pure LoS scenario. Based on the optimized PA positions obtained by the proposed SA-CDO algorithm, the corresponding waveguide radiation matrix is constructed, and the equivalent channel dictionary is computed as A=HG for sparse signal detection. Random information bits are then generated and mapped to the waveguide activation pattern and modulation symbols to construct the sparse transmit vector. After transmission through the generated channel with additive white Gaussian noise, the received signal is recovered by the corresponding detector, and the detected bits are compared with the transmitted bits to accumulate the BER statistics. For every realization, new NLoS scattering, AWGN, and transmitted bit sequences are independently generated, while the deterministic system geometry and all other simulation parameters remain unchanged. The entire procedure is repeated for each SNR point until the predefined stopping criterion is satisfied, and the reported BER is obtained by averaging over all independent channel realizations, thereby ensuring statistically fair comparisons among all considered transceiver schemes. Eight benchmark schemes are considered for performance comparison under both pure LoS and Rician fading channels: (1) *Waveguide-based IM, Fixed (Baseline)*: multi-PA waveguides as index units with uniformly fixed-PA placement, where OLS detection is employed at the receiver; (2) *Waveguide-based IM, Flexible*: baseline scheme with optimized PA positions and OLS detection; (3) *Waveguide-based IM, Fixed, OLS-CC*: baseline scheme with uniformly fixed-PA placement combined with OLS-CC detection; (4) *Waveguide-based IM, Flexible, OLS-CC*: baseline scheme with optimized PA positions combined with the proposed OLS-CC detector, corresponding to the complete proposed framework. Through controlled comparisons among these benchmark schemes, the individual contributions of PA position optimization, constellation-constrained sparse detection, and low-complexity OLS-CC detection can be quantitatively characterized, while the performance differences between pure LoS and Rician fading scenarios further demonstrate the robustness of the proposed framework under practical multipath propagation conditions.

**Figure 4:**
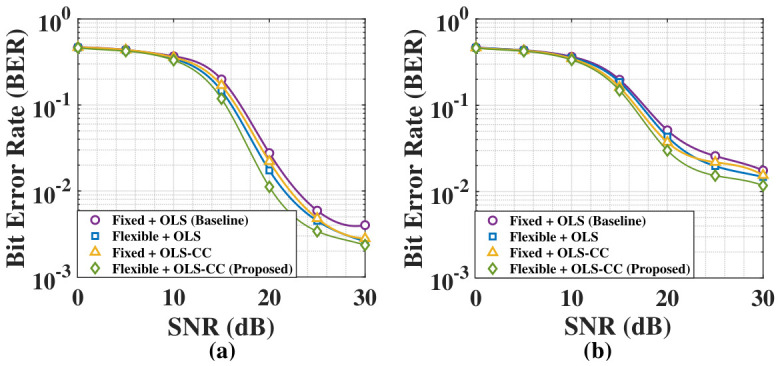
BER performance comparison of waveguide-based index modulation schemes for PASS. (**a**) Pure LoS channel; (**b**) Rician fading channel with ($K_{R}=50$).

Figure 4 compares the BER performance of four waveguide index modulation schemes under both pure LoS channels and a high-Rician-factor fading environment with $K_{R}=50$. By conducting controlled comparisons between fixed and optimized PA deployments, as well as between conventional OLS detection and the proposed OLS-CC detection, we can quantitatively separate the individual performance gains brought by PA position optimization and constellation-constrained sparse detection, and further analyze their joint synergistic benefits. The results reveal that the proposed joint framework integrating SA-CDO PA position optimization and OLS-CC detection achieves the lowest bit error rate across all tested SNR points. At SNR = 25 dB under pure LoS propagation, the Flexible+OLS-CC scheme lowers the BER from $5.9125\times 10^{-3}$ of the Fixed+OLS baseline to $3.40\times 10^{-3}$. For the $K_{R}=50$ Rician fading channel at the same SNR, the baseline BER is $2.59\times 10^{-2}$, whereas our proposed design attains a BER of $1.54\times 10^{-2}$. These observations demonstrate the capability of the SA-CDO algorithm to build a near-orthogonal equivalent channel dictionary via optimized PA placement, which effectively mitigates inter-waveguide crosstalk and boosts the detection reliability of sparse index-modulated signals. The performance gain from SA-CDO PA position optimization becomes more evident in the high-SNR region. In our simulation setup, the adopted small inter-waveguide spacing $d_{wg}=0.2\;m$ leads to strong inherent correlation between different waveguides under uniform fixed-PA layouts. In contrast, the proposed SA-CDO optimization reduces the maximum mutual coherence of the equivalent channel matrix. The measured BER data at SNR = 25 dB clearly validates this advantage: the Flexible-OLS scheme with optimized PA positions achieves a much lower error rate than the Fixed+OLS baseline. In addition, the proposed OLS-CC detector alleviates the error floor phenomenon observed in conventional OLS detection and accelerates BER decay as SNR rises. In the pure LoS scenario, the error reduction rate of standard OLS detection slows down evidently after SNR exceeds 20 dB. Benefiting from the embedded constellation constraints, the OLS-CC detector maintains steady BER decline and attains remarkably lower error rates at identical SNR values. This finding indicates that utilizing the finite-alphabet characteristic of modulation symbols can greatly refine symbol estimation accuracy and lift the overall recovery reliability of joint waveguide index and symbol information.

**Figure 5:**
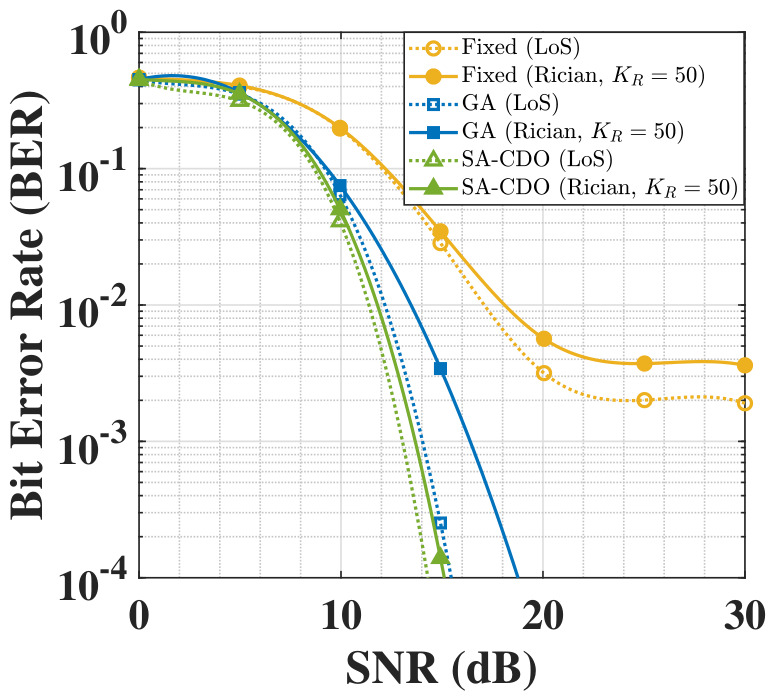
BER performance of fixed deployment, GA-optimized and the proposed SA-CDO schemes under LoS and Rician fading channels (N=12, $N_{r}=12$, $K_{R}=50$).

To further evaluate the optimization capability of the proposed SA-CDO algorithm, a benchmark comparison is conducted with a standard real-coded Genetic Algorithm (GA) [28] under a system configuration with N=12 waveguides and $N_{r}=12$ receive antennas. Both algorithms are implemented using the same optimization objective, antenna position constraints, and maximum number of objective function evaluations. In addition, the proposed OLS-CC detector is employed at the receiver for all compared schemes to ensure a fair performance comparison. The optimization quality is summarized in Table 2, where the maximum mutual coherence μ and the Gram matrix orthogonality error $∥I-A^{H}{A∥}_{F}$ are adopted as the evaluation metrics. The corresponding BER performance under both pure LoS and Rician fading channels is presented in Figure 5. As shown in Table 2, both optimization algorithms significantly improve the orthogonality of the equivalent channel dictionary compared with the conventional uniform PA deployment. Nevertheless, the proposed SA-CDO algorithm consistently achieves superior optimization performance. Specifically, compared with the GA-based optimization, SA-CDO reduces the maximum mutual coherence from 0.1849 to 0.1652, while the Gram matrix orthogonality error decreases from 2.1257 to 1.2378. These results indicate that the proposed algorithm constructs an equivalent channel dictionary with lower inter-waveguide correlation, which is more favorable for accurate sparse signal recovery at the receiver. The BER comparison shown in Figure 5 further demonstrates that the improved channel orthogonality can be effectively translated into enhanced detection performance. Under both LoS and Rician fading channels, the proposed SA-CDO algorithm consistently achieves lower BER than the GA-based optimization over the entire SNR range. These results demonstrate that, for the constrained continuous PA position optimization problem considered in this work, the proposed SA-CDO algorithm provides a more effective optimization solution than the conventional genetic algorithm.

**Figure 6:**
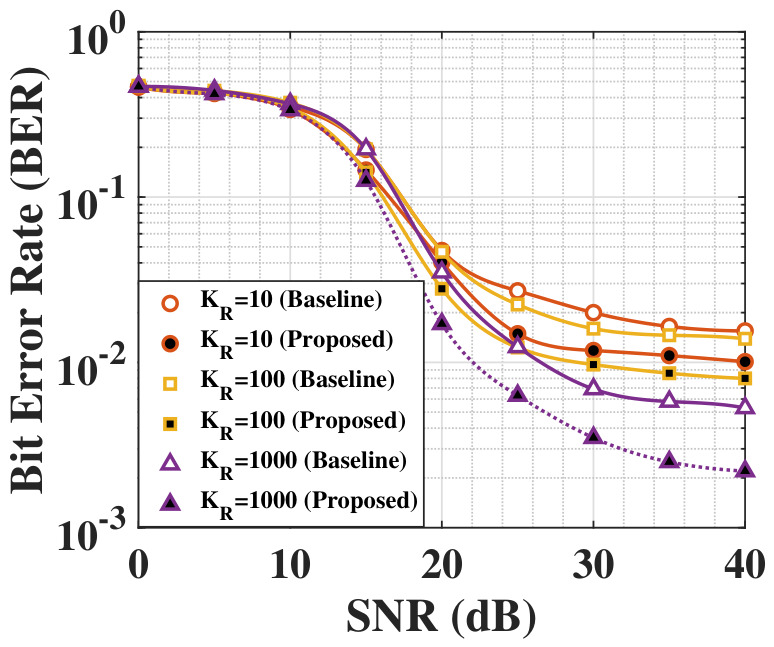
BER Performance Comparison of Baseline and Proposed Schemes under different Rician fading conditions ($K_{R}=10,100,1000$).

To further evaluate the robustness of the proposed scheme under practical IoT propagation environments, we investigate the impact of the Rician *K*-factor on the BER performance and compare the proposed scheme with the baseline under different fading conditions. Three representative Rician K-factors, namely $K_{R}=10$, 100, and 1000, are considered, corresponding to weak LoS, moderate LoS, and near-LoS propagation environments, respectively. As illustrated in Figure 6, the proposed scheme consistently achieves lower BER than the baseline over the entire SNR range for all considered Rician K-factors. Moreover, the performance advantage becomes more evident as both the SNR and the Rician K-factor increase. For example, at an SNR of 20 dB, the BER decreases from $4.75\times 10^{-2}$ to $4.04\times 10^{-2}$ for $K_{R}=10$, while for $K_{R}=100$ and 1000, the BER is further reduced to $2.79\times 10^{-2}$ and $1.71\times 10^{-2}$, respectively, compared with $4.67\times 10^{-2}$ and $3.51\times 10^{-2}$ achieved by the baseline scheme. A similar trend can be observed in the high-SNR region. At an SNR of 30 dB, the proposed scheme reduces the BER from $2.00\times 10^{-2}$ to $1.18\times 10^{-2}$ for $K_{R}=10$, corresponding to an improvement of approximately 40%. When the Rician K-factor increases to 1000, the BER is further reduced from $6.9\times 10^{-3}$ to $3.5\times 10^{-3}$, corresponding to an improvement of approximately 50%. These observations suggest that the proposed framework can provide consistent BER improvements under the considered Rician fading conditions and exhibits good robustness to different LoS propagation environments.

**Figure 7:**
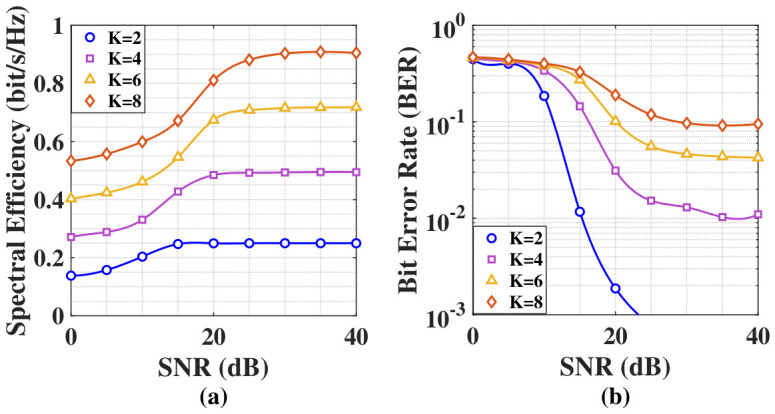
System performance under different activated waveguide numbers for the proposed scheme: (**a**) Effective spectral efficiency versus SNR; (**b**) BER versus SNR.

Figure 7a shows the effective spectral efficiency under different numbers of activated waveguides (K={2,4,6,8}). As expected, the spectral efficiency increases with the number of activated waveguides because more index combinations can be exploited to convey additional information bits without requiring extra bandwidth or transmit power. The saturation SNR gradually shifts toward higher values as *K* increases. For example, the curve with K=2 approaches its maximum spectral efficiency at approximately 20 dB, whereas the K=8 case continues to improve until around 30 dB. This behavior is attributed to the increased difficulty of jointly recovering the active waveguide indices and modulation symbols when more waveguides are activated. Nevertheless, even at an SNR of 0 dB, the proposed scheme still achieves a spectral efficiency of approximately 0.55 bit/s/Hz for K=8, demonstrating good robustness under low-SNR conditions. Figure 7b presents the corresponding BER performance. As the number of activated waveguides increases, the BER performance gradually degrades because both the index search space and the sparse support recovery complexity increase. More importantly, the equivalent codewords become more densely distributed in the signal space, resulting in a reduced minimum Euclidean distance between competing hypotheses and a higher probability of erroneous index detection. Consequently, the BER curve for K=2 exhibits a much steeper waterfall region than those for larger *K*. In contrast, when *K* increases from 2 to 4 (and beyond), the detector must jointly estimate a larger sparse support set while distinguishing among a substantially greater number of feasible index combinations. Although the proposed SA-CDO algorithm effectively suppresses the mutual coherence among waveguide channels, the increased detection ambiguity still requires a higher SNR to achieve reliable sparse support recovery, leading to a more gradual BER transition. Therefore, the observed discrepancy in the error slope mainly originates from the increased index detection complexity and the reduced effective codeword distance associated with larger numbers of activated waveguides, rather than from the proposed optimization algorithm itself. Overall, the results reveal the inherent trade-off between spectral efficiency and detection reliability. While activating more waveguides significantly improves spectral efficiency, it also increases the detection difficulty. The proposed Flexible-OLS-CC framework maintains satisfactory BER performance across all considered configurations, providing flexible operating points for different industrial IoT application requirements.

**Figure 8:**
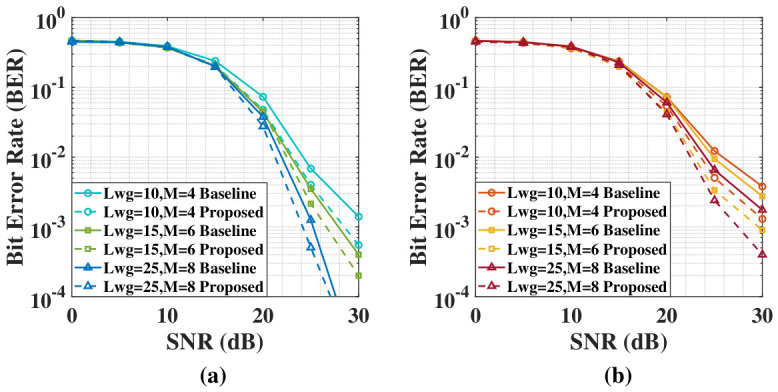
BER performance comparison of baseline and proposed schemes under different waveguide length $L_{\mathrm{wg}}$ and per-waveguide PA number *M* (fixed activated waveguide number *K*). (**a**) Pure LoS channel; (**b**) Rician fading channel with $K_{R}=50$.

Finally, Figure 8 investigates the impact of the waveguide length $L_{\mathrm{wg}}$ and the number of embedded PAs *M* on the BER performance of the proposed waveguide index modulation system. Three representative hardware configurations, namely $\left(L_{\mathrm{wg}}=10,\;M=4\right)$, $\left(L_{\mathrm{wg}}=15,\;M=6\right)$, and $\left(L_{\mathrm{wg}}=25,\;M=8\right)$, are evaluated under two propagation environments: (a) a pure LoS channel and (b) a Rician fading channel with $K_{R}=50$. For each configuration, the solid curves correspond to the Fixed-OLS benchmark with uniformly deployed PAs, while the dashed curves represent the proposed Flexible-OLS-CC scheme employing SA-CDO-based PA position optimization. As shown in both subfigures, increasing either the waveguide length or the number of embedded PAs generally improves the BER performance for both the baseline and the proposed schemes. A longer waveguide provides a larger feasible region for PA position optimization, while more embedded PAs offer additional spatial degrees of freedom for constructing a near-orthogonal equivalent channel dictionary. Consequently, the proposed scheme consistently achieves lower BER than the corresponding baseline under all considered hardware configurations. The performance improvement becomes more evident in the medium-to-high SNR region. For example, under the LoS channel at SNR=20dB, the proposed scheme reduces the BER from $1.17\times 10^{-2}$ to $7.0\times 10^{-3}$ for the $\left(L_{\mathrm{wg}}=10,\;M=4\right)$ configuration, and from $6.7\times 10^{-3}$ to $4.2\times 10^{-3}$ for the $\left(L_{\mathrm{wg}}=15,\;M=6\right)$ configuration. Similar performance improvements can also be observed under the Rician fading channel, where the BER is consistently reduced across all hardware configurations. Comparing Figure 8a,b, it can be observed that the proposed scheme maintains its performance advantage under both LoS and fading channels, indicating that the jointly designed PA position optimization and OLS-CC detection framework remains effective in the presence of multipath scattering. Overall, the results suggest that increasing the available spatial degrees of freedom through longer waveguides and more embedded PAs can further enhance the effectiveness of the proposed optimization framework while preserving robust BER performance across different propagation environments.

## 5. Conclusions

This paper proposes a waveguide-based sparse index transmission framework for PASS, a promising flexible MIMO architecture. By leveraging the unique reconfigurability of PASS-MIMO, dielectric waveguides are employed as independent index resources, and SA-CDO-based PA layout optimization is exploited to build near-orthogonal equivalent channels for inter-interference suppression. To resolve the tight coupling between PA placement and activated-index selection that leads to non-convex optimization difficulty, a two-layer transceiver design is elaborated. At the transmitter, the proposed SA-CDO algorithm optimizes PA spatial arrangement under practical physical spacing and boundary restrictions. At the receiver, an OLS-CC detector is formulated to jointly recover activated waveguide indices and modulated symbols with low computational overhead. Extensive Monte Carlo simulations verify that the proposed transceiver design surpasses traditional fixed-PA index modulation under both ideal LoS and practical $K_{R}=50$ Rician multipath fading conditions over the full tested SNR range. Benefiting from SA-CDO optimization, the system BER is remarkably reduced; meanwhile, the developed OLS-CC detector further elevates sparse detection precision and converts the original exponential detection complexity into polynomial complexity. Overall, the presented PASS-index modulation solution offers feasible design guidelines for high-reliability, scalable, flexible MIMO systems in 6G industrial IoT wireless communication scenarios.

## Figures and Tables

**Table 1:** Computational complexity comparison of different transceiver schemes.

Scheme	Transmitter (PA Optimization)	Receiver (Single-Slot Detection)
Exhaustive search + ML detection	$O(|X{|}^{N}\cdot N_{r}\left(M+N\right))$	ONK|M|KNrK
Exhaustive search + OLS detection	$O(|X{|}^{N}\cdot N_{r}\left(M+N\right))$	$O(KN_{r}N+K^{2}N_{r})$
SA-CDO + ML detection	$O(I_{\mathrm{alt}}NT_{\mathrm{total}}L_{\max}N_{r}\left(M+N\right))$	ONK|M|KNrK
SA-CDO + OLS detection	$O(I_{\mathrm{alt}}NT_{\mathrm{total}}L_{\max}N_{r}\left(M+N\right))$	$O(KN_{r}N+K^{2}N_{r})$
SA-CDO + OLS-CC (Proposed)	$O(I_{\mathrm{alt}}NT_{\mathrm{total}}L_{\max}N_{r}\left(M+N\right))$	$O\left(KN_{r}N+K^{2}N_{r}\right)+O\left(K|M|\right)$

**Table 2:** Optimization quality comparison of different PA deployment schemes.

Metric	Fixed Deployment	GA-Optimized	SA-CDO (Proposed)
Max mutual coherence μ	0.5701	0.1849	0.1652
$∥I-A^{H}{A∥}_{F}$	5.0046	2.1257	1.2378

## Data Availability

The data presented in this study are available on request from the first author.
